# Association between apoptosis inhibitor of macrophage and microsatellite instability status in colorectal cancer

**DOI:** 10.1186/s12876-020-01520-8

**Published:** 2020-11-10

**Authors:** Wen-juan Huang, Xin Wang, Meng-lin Zhang, Li Li, Rui-tao Wang

**Affiliations:** 1 Department of Internal Medicine, Harbin Medical University Cancer Hospital, Harbin Medical University, No. 150 Haping ST, Nangang District, Harbin, Heilongjiang, 150081 China; 2 Department of Colorectal Surgery, Harbin Medical University Cancer Hospital, Harbin Medical University, No. 150 Haping ST, Nangang District, HarbinHeilongjiang, 150081 China

**Keywords:** Apoptosis inhibitor of macrophage, Microsatellite instability, Colorectal cancer

## Abstract

**Background:**

The microsatellite instability (MSI) in colorectal cancer (CRC) has a more favorable clinical outcome and is characterized by highly upregulated expression of various immunological checkpoints than microsatellite stable (MSS) tumors. Apoptosis inhibitor of macrophage (AIM) is a circulating protein and circulates throughout the body to remove cellular debris. The aim of this study was to evaluate the association between MSI status and AIM levels in CRC patients.

**Methods:**

In this study, we evaluated the levels of AIM by Enzyme Linked Immuno-Sorbent Assay (ELISA) in serum of 430 CRC patients. All patients’ clinical and laboratory characteristics at initial diagnosis were collected. The relationship between AIM levels and MSI status was examined.

**Results:**

64 patients (14.9%) were identified as having MSI-H (high-frequency MSI) and 366 casess (85.1%) having MSS. Patients with an MSI-H phenotype had lower AIM levels compared with MSS patients. Moreover, AIM levels were correlated with histological type and MSI status. Logistic regression analysis revealed that decreased AIM levels were independently associated with MSI-H phenotype after adjusting confounding factors.

**Conclusion:**

Reduced AIM levels are associated with MSI-H subtyping of CRC. Further research on the involvement of AIM in MSI-H CRC is needed.

## Background

Colorectal cancer (CRC) was the third most common malignant cancer and the fourth leading cause of cancer-related deaths worldwide [1]. Despite the advancement in comprehensive treatment, the long-term survival of CRC patients remains unsatisfactory. More than 20% of CRC patients were diagnosed with distant metastasis at initial diagnosis [2]. The microsatellite instability (MSI) subtype of CRC accounts for approximately 15% of colorectal cancers and results from the accumulation of frameshift mutations in target gene caused by a failure of the mismatch repair system [3]. MSI CRCs exhibit proximal colonic location, increased lymphocytic infiltration, and poorer response to chemotherapeutic drugs, and are characterized by highly upregulated expression of various immunological checkpoints [4, 5].

Apoptosis inhibitor of macrophage (AIM) is a circulating protein of approximately 40 kDa and mainly produced by tissue-resident macrophages, including liver Kupffer cells and peritoneal macrophages [6]. AIM, a member of the scavenger receptor cysteine-rich superfamily, is discharged from a macrophage and circulates throughout the body to remove cellular debris [7]. Moreover, recent studies revealed that AIM plays key roles in lipid accumulation, acute kidney injury, acute myocardial infarction, acute lung injury, sepsis, hepatic fibrosis, and hepatocellular carcinoma [8–15].

However, there are few studies to evaluate AIM levels in patients with MSI CRCs. Therefore, the aim of this study was to compare MSI status with AIM levels in patients with CRC.

## Methods

### Study population

We studied 430 patients with CRC at the Harbin Medical University Cancer Hospital between January 2018 and December 2018. All patients were confirmed by histology. None of CRC patients received chemotherapy or radiotherapy. The exclusion criteria included hematological disorders, hypertension, and diabetes mellitus.

This study was approved by the Institutional Review Board of Harbin Medical University Cancer Hospital. All of the patients provided their written consent to participate in the study.

### Clinical examination and biochemical measurements

Clinical and demographic characteristics of all patients were recorded, including smoking status, drinking status, medical history and medication use. A venous blood sample was collected in anticoagulant-free tube from each participant under fasting conditions prior to any treatment. The blood samples were centrifuged at 2500*g* for 10 min and the serum was then stored at − 80 °C. Routine blood tests were conducted in the hospital’s clinical laboratory.

### ELISA measurements

AIM was measured using a commercially available sandwich ELISA (*CUSABIO*, Wuhan, China) according to the recommendation of the manufacturer. Samples were measured as duplicates. The intra- and inter assay variation were below 8%.

### MSI analysis

DNA was obtained from fresh-frozen tumor tissue samples. MSI was assessed using polymerase chain reaction with primers amplifying the microsatellite markers, including BAT25, BAT26, NR-21, NR-24, and NR-27. MSI was graded as high (MSI-H) if at least three markers out of five were unstable, whereas MSS was defined as stable (MSS) if there were less than three unstable markers. There were no samples with only two unstable markers.

### Statistical analysis

All data were presented as means ± standard deviation or median (interquartile range) for continuous variables and percentages of the number for categorical variables. Normally distributed continuous variables in two groups were compared with the Student’s t test and skewed-distributed with the Mann–Whitney *U* test. The chi-square test was used for categorical variables. Logistic regression analysis was carried out to evaluate clinicopathological factors that were associated with MSI-H status. The statistical analyses were performed using SPSS Statistics version 25.0 (SPSS Inc., Chicago, IL, USA). Receiver-operating characteristics (ROC) curve analysis was used to identify cut-off value of AIM using MedCalc version 15.0. A two-tailed *P* < 0.05 indicated statistical significance.

## Results

The study included 430 CRC patients between January 2018 and December 2018. Of the 430 participants entered, 245 (57.0%) were men and 185 (43.0%) were women. The mean ages were 59.5 ± 9.9 and 59.2 ± 10.2 years, respectively.

The characteristics of CRC patients are summarized according to MSI status in Table 1. There were no significant differences in gender, smoking status, drinking status, and creatinine levels between the two groups. CRC patients with MSI-H were young and had higher BMI, WBC, platelet count, AIM levels, and lower CEA and haemoglobin levels, compared to the patients with MSS.

**Table 1:** Clinical and laboratory characteristics of the participants according to MSI status

Variables	MSI-H	MSS	P value
Number	64	366	
Age (years)	59.4 ± 11.8	61.0 ± 9.4	0.328
Gender (female, %)	33 (51.6)	148 (40.4)	0.096
BMI (kg/m^2^)	24.5 ± 3.3	23.2 ± 3.2	0.004
Current smoker (%)	25 (39.1)	158 (43.2)	0.540
Drinker (n, %)	17 (26.6)	122 (33.3)	0.285
Creatinine (μmol/L)	80.9 ± 19.7	81.1 ± 18.4	0.929
CEA (ng/mL)	3.14 (1.57–7.98)	4.86 (2.15–12.12)	0.030
WBC (× 10^9^/L)	8.07 ± 2.98	6.92 ± 2.30	0.004
Haemoglobin (g/L)	124.6 ± 27.3	134.3 ± 22.4	0.009
Platelet count (× 10^9^/L)	309.6 ± 121.8	266.8 ± 82.6	0.008
AIM (μg/mL)	5.89 (1.37)	6.86 (1.49)	< 0.001

Data are presented as means (SD) or median (interquartile range) or percentage

*BMI* body mass index, *CEA* carcinoembryonic antigen, *WBC* white blood cells, *AIM* apoptosis inhibitor of macrophage

The association between clinicopathological features and MSI status in CRC patients is shown in Table 2. There were significantly positive correlations between MSI-H status and tumor location, tumor size, histological grade, lymphatic invasion, lymph node metastasis, clinical stage, and histological type. However, no correlations were found between MSI-H and T classification, and distant metastasis.

**Table 2:** Correlations between clinicopathological features and MSI status in CRC

Variables	Total	MSI-H	MSS	P value
	n (%)	n (%)	n (%)	
*Tumor location*				< 0.001
Proximal	153 (35.6)	41 (64.1)	112 (30.6)	
Distal	277 (64.4)	23 (35.9)	254 (69.4)	
*Tumor size (cm)*				0.011
< 5.0	282 (65.6)	33 (51.6)	249 (68.0)	
≥ 5.0	148 (34.4)	31 (48.4)	117 (32.0)	
*Morphological type*				0.717
Expansive	155 (36.0)	21 (32.8)	134 (36.6)	
Infiltrative	20 (4.7)	4 (6.3)	16 (4.4)	
Ulcerative	255 (59.3)	39 (60.9)	216 (59.0)	
*Histological type*				< 0.001
Non-mucinous	364 (84.7)	42 (65.6)	322 (88.0)	
Mucinous	66 (15.3)	22 (34.4)	44 (12.0)	
*Histological grade*				0.008
Well/moderately differentiated	308 (71.6)	37 (57.8)	271 (74.0)	
Poorly differentiated	122 (28.4)	27 (42.2)	95 (26.0)	
*Lymphatic invasion*				0.030
Absent	331 (77.0)	56 (87.5)	275 (75.1)	
Present	99 (23.0)	8 (12.5)	91 (24.9)	
*T classification *				0.811
T1 + T2	63 (14.7)	10 (15.6)	53 (14.5)	
T3 + T4	367 (85.3)	54 (84.4)	313 (85.5)	
*Lymph node metastasis*				0.010
Absence	267 (62.1)	49 (76.6)	218 (59.6)	
Presence	163 (37.9)	15 (23.4)	148 (40.4)	
*Distant metastasis*				0.713
Absence	382 (88.8)	56 (87.5)	326 (89.1)	
Presence	48 (11.2)	8 (12.5)	40 (10.9)	
*Stage *				0.003
I–II	258 (60.0)	49 (76.6)	209 (57.1)	
III–IV	172 (40.0)	15 (23.4)	157 (42.9)	

**Fig. 1:**
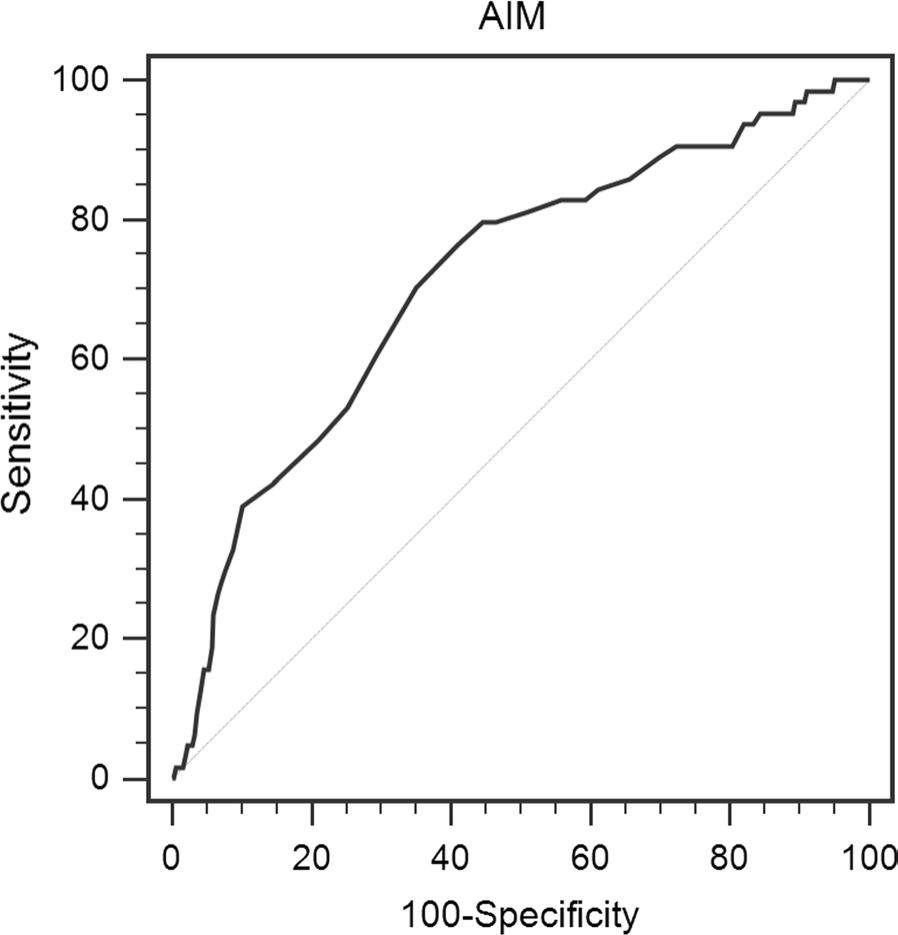
Optimal cut-off value was determined for AIM using standard ROC curve analysis

The median value of AIM was 6.8 μg/mL (range 3.6–12.3 μg/mL). ROC analysis was used to assess the optimal cutoff value for AIM was 6.3 for MSI-H phenotype (AUC = 0.715, 95% CI 0.645–0.783, p < 0.001) (Fig. 1). CRC patients were divided into two groups according to the cutoff level. Of the total of 430 patients, 199 patients (46.3%) were detected with AIM of less than or equal to 6.3 μg/mL, while there were 231 patients (53.7%) whose AIM levels were greater than 6.3 μg/mL. Correlations between AIM and clinicopathologic variables are presented in Table 3. There were significant differences in age, histological type, and MSI status. However, gender, BMI, smoking status, drinking status, WBC, haemoglobin, tumor size, tumor location, lymphatic invasion, venous invasion, T classification, lymph node metastasis, distant metastasis, and clinical stage in two groups did not show significant differences.

**Table 3:** Baseline characteristics of CRC patients according to AIM levels

Variables	Total	AIM ≤ 6.3	AIM > 6.3	*P*
Age (years)				0.002
≤ 65	282 (65.6)	115 (57.8)	167 (72.3)	
> 65	148 (34.4)	84 (42.2)	64 (27.7)	
Gender				0.787
Male	245 (57.0)	112 (56.3)	133 (57.6)	
Female	185 (43.0)	87 (43.7)	98 (42.4)	
BMI (kg/m^2^)	23.4 ± 3.2	23.6 ± 3.4	23.3 ± 3.0	0.333
Current smoker				0.359
Yes	183 (42.6)	80 (40.2)	103 (44.6)	
No	247 (57.4)	119 (59.8)	128 (55.4)	
Drinker				0.371
Yes	139 (32.3)	60 (30.2)	79 (34.2)	
No	291 (67.7)	139 (69.8)	152 (65.8)	
WBC (× 10^9^/L)	7.09 ± 2.44	7.11 ± 2.61	7.08 ± 2.30	0.923
Haemoglobin (g/L)	132.9 ± 23.4	130.8 ± 23.7	134.7 ± 23.1	0.086
Platelet count (× 10^9^/L)	273.1 ± 90.6	272.6 ± 89.7	273.6 ± 91.6	0.914
Creatinine (μmol/L)	81.1 ± 18.6	82.6 ± 22.3	79.8 ± 14.7	0.132
CEA (ng/mL)	4.37 (2.03–11.31)	3.82 (1.94–13.70)	4.67 (2.12–10.67)	0.768
Tumor size (cm)				0.738
< 5.0	278 (64.7)	127 (63.8)	151 (65.4)	
≥ 5.0	152 (35.3)	72 (36.2)	80 (34.6)	
Tumor location				0.063
Proximal	153 (35.6)	80 (59.8)	73 (31.6)	
Distal	277 (64.4)	119 (31.2)	158 (68.4)	
Histological type				0.045
Non-mucinous	364 (84.7)	161 (80.9)	203 (87.9)	
Mucinous	66 (15.3)	38 (19.1)	28 (12.1)	
Histological grade				0.598
Well/moderately differentiated	308 (71.6)	145 (72.9)	163 (70.6)	
Poorly differentiated	122 (28.4)	54 (27.1)	68 (29.4)	
Lymphatic invasion				0.381
Absent	331 (77.0)	157 (78.9)	174 (75.3)	
Present	99 (23.0)	42 (21.1)	57 (24.7)	
T classification				0.061
T1 + T2	63 (14.7)	36 (18.1)	27 (11.7)	
T3 + T4	367 (85.3)	163 (81.9)	204 (88.3)	
Lymph node metastasis				0.377
Absence	267 (62.1)	128 (64.3)	139 (60.2)	
Presence	163 (37.9)	71 (35.7)	92 (39.8)	
Distant metastasis				0.709
Absence	382 (88.8)	178 (89.4)	204 (88.3)	
Presence	48 (11.2)	21 (10.6)	27 (11.7)	
Stage				0.134
I–II	258 (60.0)	127 (63.8)	131 (56.7)	
III–IV	172 (40.0)	72 (36.2)	100 (43.3)	
MSI status				< 0.001
MSS	366 (85.1)	150 (75.4)	216 (93.5)	
MSI-H	64 (14.9)	49 (24.6)	15 (6.5)	

*BMI* body mass index, *WBC* white blood cells, *AIM* apoptosis inhibitor of macrophage

**Fig. 2:**
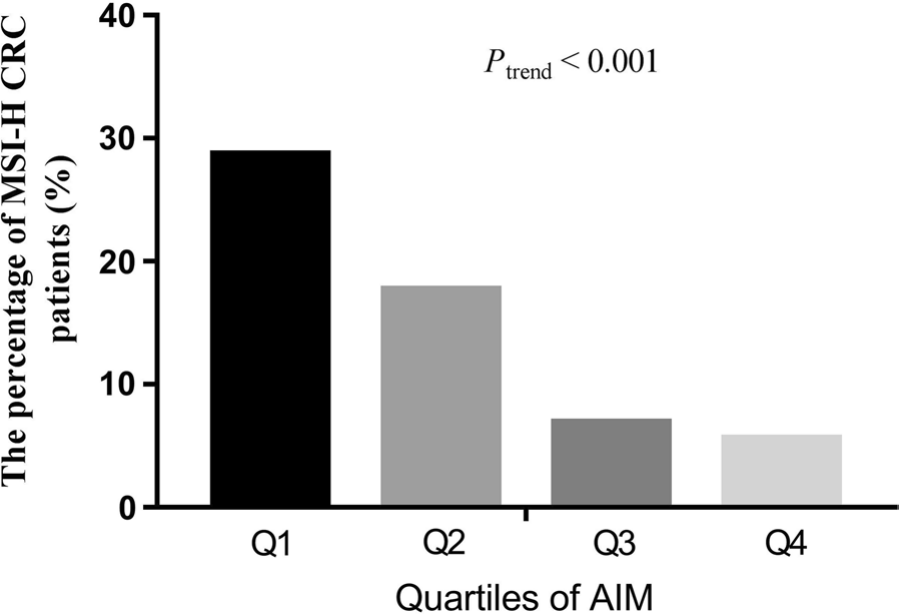
The association between the percentages of MSI-H CRC and AIM levels

All CRC patients were classified into quartiles according to their AIM levels, including quartile 1 (Q1) ≤ 5.9 µg/mL, 5.9 µg/mL < quartile 2 (Q2) ≤ 6.5 µg/mL, 6.5 µg/mL < quartile 3 (Q3) ≤ 7.2 µg/mL, and quartile 4 (Q4) ≥ 7.3 µg/mL (Fig. 2). The percentages of patients with MSI-H in each group were 28.7%, 17.7%, 6.9% and 5.6%, respectively. The results showed that as the serum AIM levels increased, the percentage of patients with MSI-H reduced.

Logistic regression analysis was performed to evaluate the clinicopathological factors that were associated with MSI-H status. Fourteen variables, including gender, BMI, WBC, haemoglobin, platelet count, CEA, AIM, tumor size, tumor location, lymphatic invasion, histological grade, histological type, lymph node metastasis, and clinical stage, were entered into the original equation. The factors found to be significantly associated with MSI-H in the regression analysis included BMI, WBC, platelet count, AIM, tumor size, tumor location, histological type, and histological grade (Table 4). Notably, reduced AIM levels were associated with a higher risk of MSI-H phenotype after adjusting for other confounding variables.

**Table 4:** Logistic regression analysis to evaluate the associations between MSI status and clinical factors

Variables	β	OR (95% CI)	P value
Gender (female vs male)	0.520	1.682 (0.842–3.360)	0.141
BMI (kg/m^2^)	0.158	1.171 (1.054–1.301)	0.003
WBC (× 10^9^/L)	0.142	1.152 (1.014–1.310)	0.030
Haemoglobin (g/L)	0.000	1.000 (0.985–1.014)	0.957
Platelet count (× 10^9^/L)	0.005	1.005 (1.001–1.009)	0.021
CEA (ng/ml)	− 0.004	0.996 (0.985–1.008)	0.549
AIM (μg/mL)	− 0.646	0.524 (0.375–0.731)	< 0.001
Tumor size (cm)			
(≥ 5.0 vs < 5.0)	1.178	3.249 (1.631–6.474)	0.001
Tumor location			
(Proximal vs distal)	1.203	3.330 (1.642–6.752)	0.001
Histological type			
(Mucinous vs non-mucinous)	1.452	4.273 (1.974–9.248)	< 0.001
Histological grade			
(Poorly differentiated vs well/moderately differentiated)	1.361	3.900 (1.881–8.086)	< 0.001
Lymphatic invasion			
(Presence vs absence)	− 0.695	0.499 (0.186–1.339)	0.168
Lymph node metastasis			
(Presence vs absence)	0.629	1.875 (0.188–18.671)	0.592
Stage			
(III + IV vs I + II)	− 1.863	0.155 (0.015–1.600)	0.118

Data are presented as means (SD) or median (interquartile range) or percentage

*BMI* body mass index, *WBC* white blood cells, *AIM* apoptosis inhibitor of macrophage

## Discussion

It has been shown for the first time that AIM levels were significantly reduced in MSI-H CRC patients compared with those in MSS CRC patients. AIM levels were correlated with age, histological type, and MSI status. Moreover, AIM levels were independently associated with MSI-H phenotype.

Macrophages in tumor microenvironment play a vital role in tumor development, angiogenesis, and metastasis [16, 17]. Recent studies confirmed that macrophages modulates the immune response against pathogens and maintains tissue homeostasis in cancer [18]. The main chemokines secreted from cancer cells attract macrophage and promote the expansion and dissemination of cancer cells [19]. Many reports demonstrated that tumor associated macrophages (TAMs) are one of the key targets to improve the efficacy of immunotherapies as these cells can suppress the functions of CD8^+^ T and NK cells [20]. M2-like TAMs are thought to drive neoangiogenesis, suppress the adaptive immune response, and promote tumor cell proliferation, invasion, and metastasis [18]. High TAM density was associated with worse survival in oral cancer, breast cancer, gastric cancer, bladder cancer, and ovarian cancer [20]. However, high TAM density has a better survival in CRC patients with or without metastases [21–23].

The exact mechanisms of AIM in MSI-H CRCs were currently unclear. AIM was recognized to have an apoptosis inhibitory function for macrophages, T cells, and natural killer T cells [24]. However, AIM had different effects in different cancers. AIM overexpression in myeloid cells led to the formation of lung adenocarcinoma in a transgenic mouse model [25]. However, AIM-deficient mice were highly susceptible to steatosis-associated the development of hepatocellular carcinoma [26]. The difference of AIM in different CRC subtypes supports the crucial roles that macrophages play on immune cells. AIM acts as a marker for phagocytes so that they can efficiently recognize and engulf the debris as their target [7]. Moreover, the phagocytic activities performed by the non-professional phagocytes contribute to the physiological tissue turnover or remodeling, leading to maintenance of the tissue homeostasis. AIM plays a crucial role in obesity-induced inflammation in white adipose tissue as characterized by decreased proinflammatory M1 macrophages but increased anti-inflammatory M2 macrophages [6]. A recent report revealed that glycogen synthase kinase 3 modulates obesity-induced visceral adipose tissue inflammation by inhibiting AIM production in macrophages [27].

Consistent to our results, previous studies showed that the CD8 T effector gene signature was significantly upregulated in MSI-H tumors compared with MSI-L/MSS tumors [28]. Moreover, a report demonstrated that CD8(+) cytotoxic T lymphocytes may lead to increased platelet destruction in immune thrombocytopenia [29].

In this study, we found that activated platelets are involved in different CRC subtypes. Therefore, investigating the mechanism of AIM involved in MSI-H CRCs may be helpful for guiding treatment strategy in different CRC subtypes.

Some limitations of the present study need to be acknowledged: First, the study was performed in a single hospital. Second, our data can not provide a mechanistic explanation for our findings. Third, the results cannot be generalized because the study included only Chinese patients.

## Conclusions

AIM levels were decreased in MSI-H CRCs. Further mechanistic research was needed.

## Data Availability

The data in this study available from the corresponding author on reasonable request.

## Abbreviations

AIM: Apoptosis inhibitor of macrophage
CRC: Colorectal cancer
MSI: Microsatellite instability
MSS: Microsatellite stable
